# Supplementary material for: Multi‐dataset identification of innovative feature genes and molecular mechanisms in keratoconus
Source: J Cell Mol Med. 2024 Sep 19;28(18):e70079. doi: 10.1111/jcmm.70079 (PMC11412914; doi:10.1111/jcmm.70079)
Supplement: Supplementary file 2 — Material S2. [file JCMM-28-e70079-s002.zip › 7.GSEA analysis/4.MYRF/KEGG_GLYCOSAMINOGLYCAN_BIOSYNTHESIS_HEPARAN_SULFATE.html]

Details for gene set KEGG\_GLYCOSAMINOGLYCAN\_BIOSYNTHESIS\_HEPARAN\_SULFATE[GSEA]

|  || Dataset | MYRF.group.cls#h\_versus\_l |
| Phenotype | group.cls#h\_versus\_l |
| Upregulated in class | h |
| GeneSet | KEGG\_GLYCOSAMINOGLYCAN\_BIOSYNTHESIS\_HEPARAN\_SULFATE |
| Enrichment Score (ES) | 0.3026265 |
| Normalized Enrichment Score (NES) | 1.0209045 |
| Nominal p-value | 0.40361446 |
| FDR q-value | 1.0 |
| FWER p-Value | 1.0 |
Table: GSEA Results Summary

  

Fig 1: Enrichment plot: KEGG\_GLYCOSAMINOGLYCAN\_BIOSYNTHESIS\_HEPARAN\_SULFATE      
 Profile of the Running ES Score & Positions of GeneSet Members on the Rank Ordered List

  

| SYMBOL | TITLE | RANK IN GENE LIST | RANK METRIC SCORE | RUNNING ES | CORE ENRICHMENT || 1 | HS3ST3A1 | na | 567 | 0.556 | 0.1376 | Yes |
| 2 | NDST4 | na | 3031 | 0.293 | 0.1477 | Yes |
| 3 | NDST1 | na | 4235 | 0.246 | 0.1812 | Yes |
| 4 | HS6ST3 | na | 4974 | 0.223 | 0.2217 | Yes |
| 5 | HS3ST2 | na | 5303 | 0.213 | 0.2713 | Yes |
| 6 | EXTL1 | na | 6743 | 0.171 | 0.2773 | Yes |
| 7 | HS3ST3B1 | na | 7349 | 0.154 | 0.3026 | Yes |
| 8 | HS3ST5 | na | 9843 | 0.099 | 0.2581 | No |
| 9 | B3GALT6 | na | 11357 | 0.073 | 0.2346 | No |
| 10 | EXTL3 | na | 13410 | 0.045 | 0.1879 | No |
| 11 | XYLT1 | na | 14200 | 0.036 | 0.1750 | No |
| 12 | HS6ST1 | na | 15288 | 0.025 | 0.1506 | No |
| 13 | B3GAT1 | na | 18510 | -0.005 | 0.0592 | No |
| 14 | NDST2 | na | 18911 | -0.009 | 0.0503 | No |
| 15 | XYLT2 | na | 20667 | -0.030 | 0.0079 | No |
| 16 | HS6ST2 | na | 21030 | -0.034 | 0.0069 | No |
| 17 | B3GAT3 | na | 22420 | -0.051 | -0.0190 | No |
| 18 | EXT2 | na | 22570 | -0.053 | -0.0086 | No |
| 19 | B4GALT7 | na | 22839 | -0.057 | -0.0005 | No |
| 20 | HS2ST1 | na | 23477 | -0.067 | -0.0003 | No |
| 21 | EXT1 | na | 23764 | -0.072 | 0.0114 | No |
| 22 | EXTL2 | na | 25248 | -0.098 | -0.0042 | No |
| 23 | B3GAT2 | na | 26655 | -0.127 | -0.0096 | No |
| 24 | GLCE | na | 28416 | -0.173 | -0.0124 | No |
| 25 | HS3ST1 | na | 29241 | -0.198 | 0.0188 | No |
| 26 | NDST3 | na | 33893 | -0.503 | 0.0241 | No |
Table: GSEA details [plain text format]

  

Fig 2: KEGG\_GLYCOSAMINOGLYCAN\_BIOSYNTHESIS\_HEPARAN\_SULFATE      
 Blue-Pink O' Gram in the Space of the Analyzed GeneSet

  

Fig 3: KEGG\_GLYCOSAMINOGLYCAN\_BIOSYNTHESIS\_HEPARAN\_SULFATE: Random ES distribution      
 Gene set null distribution of ES for **KEGG\_GLYCOSAMINOGLYCAN\_BIOSYNTHESIS\_HEPARAN\_SULFATE**
